# 2-Bromo-5-*tert*-butyl-*N*-methyl-*N*-[2-(methyl­amino)­phen­yl]-3-(1-methyl-1*H*-benzimidazol-2-yl)benzamide

**DOI:** 10.1107/S1600536814014433

**Published:** 2014-06-25

**Authors:** Poonam Rajesh Prasad, Shikha Das, Harkesh B. Singh, Ray J. Butcher

**Affiliations:** a Department of Chemistry, Indian Institute of Technology Bombay, Powai, Mumbai 400 076, India; b Department of Chemistry, Howard University, 525 College Street NW, Washington, DC 20059, USA

**Keywords:** crystal structure

## Abstract

In the title compound, C_27_H_29_BrN_4_O, benzimidazole ring system and the amide moiety are planar [r.m.s. deviations = 0.016 (2) and 0.017 (1) Å, respectively]. The mol­ecule adopts a conformation in which the amide linkage is almost perpendicular to the central ring [dihedral angle = 85.79 (8)°], while the benzimidazole ring system makes a dihedral angle of 70.26 (11)° with the central ring. In the crystal, the mol­ecules form dimers through N—H⋯O hydrogen bonds and C—H⋯O interactions. These dimers are further linked into zigzag ribbons along [201] by weak C—H⋯Br inter­actions. As a result of the bulky nature of the mol­ecule, as evidenced by the large dihedral angles between rings, there is little evidence for any π–π inter­actions.

## Related literature   

The metal binding properties of imidazole-containing pincer ligands can be modified by the type of donor atoms and the electron-withdrawing and electron-releasing character of their substituents, see: Selander & Szabó (2011 ▶). For the effect of *N*-substitution on the catalytic activity of phosphinoimidazolines in palladium-catalysed Heck reactions, see: Busacca *et al.* (2003 ▶). For the use of bromine-substituted benzimidazole in Heck reactions, see: Reddy & Krishna (2005 ▶). For standard bond lengths, see: Allen *et al.* (1987 ▶). For the preparation of the precursor, 2-bromo-5-(*tert*-but­yl)isophthalic acid, see: Field *et al.* (2003 ▶).
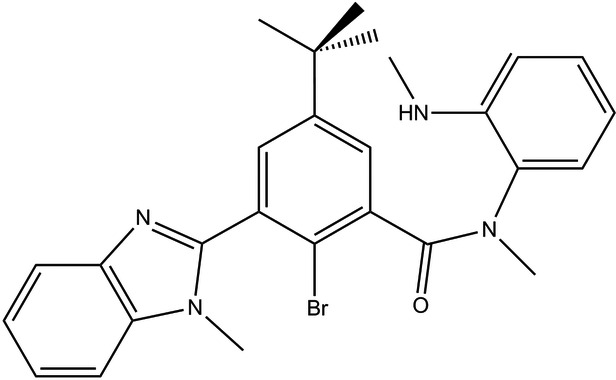


## Experimental   

### 

#### Crystal data  

C_27_H_29_BrN_4_O
*M* *_r_* = 505.45
Monoclinic, 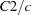

*a* = 34.4327 (13) Å
*b* = 9.4152 (2) Å
*c* = 17.1092 (7) Å
β = 118.312 (5)°
*V* = 4883.2 (3) Å^3^
*Z* = 8
Cu *K*α radiation
μ = 2.50 mm^−1^
*T* = 123 K
0.38 × 0.32 × 0.23 mm

#### Data collection  

Agilent Xcalibur Ruby Gemini diffractometer
Absorption correction: multi-scan (*CrysAlis PRO*; Agilent, 2012 ▶) *T*_min_ = 0.788, *T*_max_ = 1.000
9307 measured reflections
4929 independent reflections
4100 reflections with *I* > 2σ(*I*)
*R*_int_ = 0.028

#### Refinement  

*R*[*F* ^2^ > 2σ(*F* ^2^)] = 0.034
*wR*(*F* ^2^) = 0.093
*S* = 1.03
4929 reflections
308 parameters
H atoms treated by a mixture of independent and constrained refinement
Δρ_max_ = 0.43 e Å^−3^
Δρ_min_ = −0.35 e Å^−3^

### 

Data collection: *CrysAlis PRO* (Agilent, 2012 ▶); cell refinement: *CrysAlis PRO*; data reduction: *CrysAlis PRO*; program(s) used to solve structure: *SHELXS97* (Sheldrick, 2008 ▶); program(s) used to refine structure: *SHELXL97* (Sheldrick, 2008 ▶); molecular graphics: *SHELXTL* (Sheldrick, 2008 ▶); software used to prepare material for publication: *SHELXTL*.

## Supplementary Material

Crystal structure: contains datablock(s) I. DOI: 10.1107/S1600536814014433/jj2190sup1.cif

Structure factors: contains datablock(s) I. DOI: 10.1107/S1600536814014433/jj2190Isup2.hkl

Supporting information file. DOI: 10.1107/S1600536814014433/jj2190Isup3.cml

CCDC reference: 1009070

Additional supporting information:  crystallographic information; 3D view; checkCIF report

## Figures and Tables

**Table 1:** Hydrogen-bond geometry (Å, °)

*D*—H⋯*A*	*D*—H	H⋯*A*	*D*⋯*A*	*D*—H⋯*A*
N3—H3*B*⋯O1^i^	0.81 (3)	2.35 (3)	3.038 (3)	143 (3)
C4—H4*A*⋯Br^ii^	0.95	2.98	3.719 (3)	135
C12—H12*A*⋯O1^i^	0.95	2.37	3.287 (3)	163

Symmetry codes: (i) 
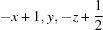
; (ii) 
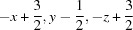
.

**Fig. 1.**
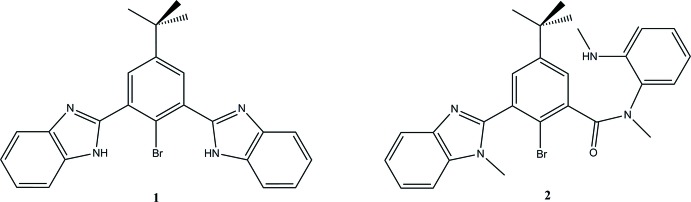
The structures of 1 and 2.

**Fig. 2.**
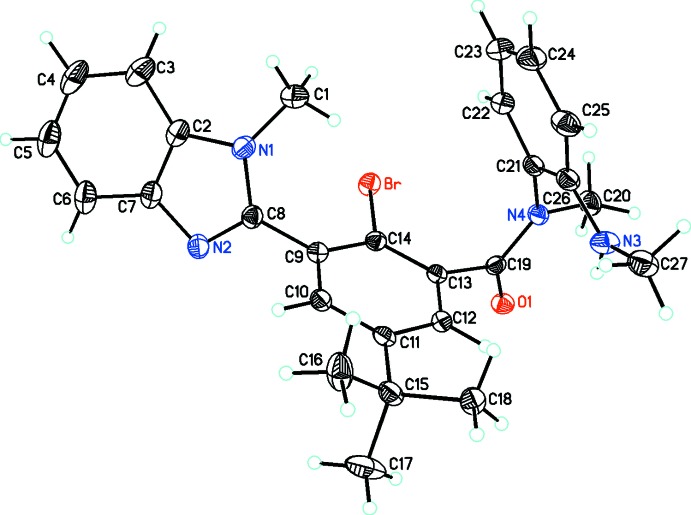
The molecular structure of C27H29BrN4O, showing the atom numbering scheme and 30% probability displacement ellipsoids and the linking of the molecules into dimers by N—H···O hydrogen bonds (shown as dashed bonds).

**Fig. 3.**
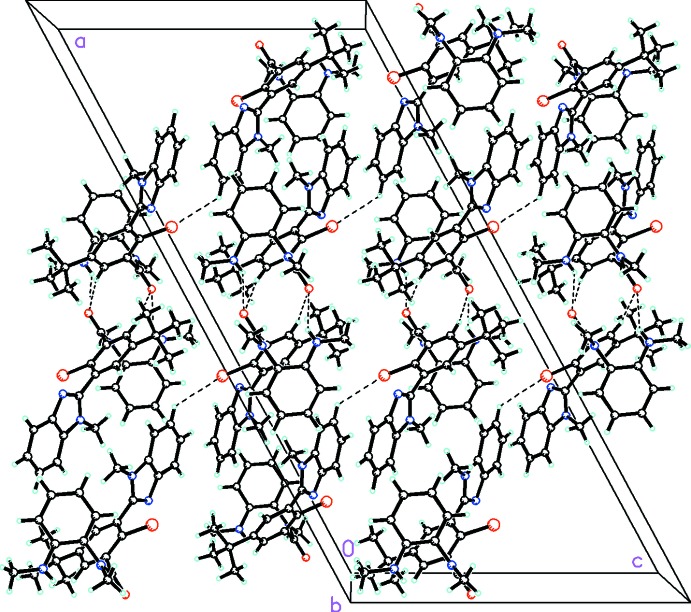
The molecular packing for C27H29BrN4O viewed along the b axis showing linking of the hydrogen bonded dimers into zigzag chains in the [2 0 1] direction by C—H···Br interactions (N—H···O and C—H···Br interactions shown as dashed bonds).

## supplementary crystallographic information

### S1. Experimental

The methyl­ation reaction of 2 (Fig. 1) was carried out by reacting 1 (0.5 g, 1.12 mmol) with an excess of methyl iodide (1.75 g, 10 eq), followed by the addition of KOH (0.125 g, 2.24 mmol) in dry acetone (20 mL) and some molecular sieves. The reaction mixture was refluxed for 2 h. Then, it was diluted with ethyl acetate and washed with water. The organic layer was dried over Na_2_SO_4_ and purified by column chromatography to afford 2 which was crystallized from a mixture of di­chloro­methane and ether. Anal. Calcd. for C_27_H_29_BrON_4_: C, 64.16; H, 5.78; N, 11.08; found C, 64.30; H, 6.22; N, 9.17.

### S1.1. Refinement

H atoms were placed in geometrically idealized positions and constrained to ride on their parent atoms with a C—H distances of 0.95 and 0.98 Å *U*_iso_(H) = 1.2*U*_eq_(C) and 0.96 Å for CH_3_ [*U*_iso_(H) = 1.5*U*_eq_(C)]. The hydrogen atom attached to N3 was located in a difference Fourier and refined isotropically.

### S2. Comment

The presence of imidazole rings in any molecular framework provides excellent modification sites for the fine tuning of properties related to electronic and steric factors. It has been reported in the literature that the strong electronic effect can be modified by the type of donor atoms and the electron-withdrawing and electron-releasing character of their substituents (Selander, & Szabó, 2011). Recently the effect of *N*-substitution on the catalytic activities of phosphinoimidazolines in palladium catalyzed Heck reactions has been reported (Busacca *et al.*, 2003). Later, Reddy and co-workers (Reddy, & Krishna, 2005) have studied the use of bromine substituted benzimidazole in Heck reactions. Pincer ligands have immense scope in exploring different types of metal coordination chemistry and stabilizing unusual species. They provide the sites which can be easily fine tuned to synthesize a number of metal complexes/species, which can be stabilized by three coordinating/bonding units of the pincer ligands. There are no examples of selenium containing benzimidazoles known in the literature. Therefore, 2, 2'-(2-bromo-5-(*tert*-butyl)-1,3-diyl)bis­(1H-benzimidazole) (1) and its derivatives, having two coordinating imidazole rings were designed to incorporate selenium at 2*-*position of the phenyl group. An attempted methyl­ation of 1 led to cleavage of the one of the benzimidazole rings and resulted in the formation of unexpected compound 2 (Fig. 1). 2-Bromo-5-(*tert*-butyl)­isophthalic acid, the precursor for synthesizing 1, was prepared according to literature procedure (Field, *et al.*, 2003). Compound 1 was synthesized by the reaction of 2-bromo-5-*tert*-butyl-isophthalic acid with 1,2-phenyl­enedi­amine in polyphospho­ric acid at 240°C.

In view of the above, the structure of the title compound, C_27_H_29_BrN_4_O, was determined (Fig. 2). The bond lengths and angles are all in the expected ranges (Allen *et al.*, 1987) for such compounds. All the aromatic groups and the amide moiety are planar (rms deviations of 0.006 (1), 0.008 (2), 0.016 (2), and 0.017 (1) for the central phenyl ring, the substituent phenyl ring, the benzimidazole ring, and the amide moiety, respectively). The molecule adopts a conformation where the amide linkage is almost perpendicular to the central ring with a dihedral angle of 85.79 (8)° between central ring (C9–C14) and amide moiety (C19/C20/C21/N4/O1) while the benzimidazole ring makes a dihedral angle of 70.26° with the central ring. The molecules form dimers through N3—H···O1 inter­molecular hydrogen bonds (Fig. 3). These dimers are further linked into zig-zag ribbons in the [2 0 1] direction by weak C—H···Br inter­actions. Because of the bulky nature of the molecule, as evidenced by the large dihedral angles between rings, there is little evidence for any π–π inter­actions.

### Crystal data

**Table:**

C_27_H_29_BrN_4_O	*F*(000) = 2096
*M**_r_* = 505.45	*D*_x_ = 1.375 Mg m^−^^3^
Monoclinic, *C*2/*c*	Cu *K*α radiation, λ = 1.54184 Å
Hall symbol: -C 2yc	Cell parameters from 4036 reflections
*a* = 34.4327 (13) Å	θ = 2.9–75.5°
*b* = 9.4152 (2) Å	µ = 2.50 mm^−^^1^
*c* = 17.1092 (7) Å	*T* = 123 K
β = 118.312 (5)°	Prism, colorless
*V* = 4883.2 (3) Å^3^	0.38 × 0.32 × 0.23 mm
*Z* = 8	

### Data collection

**Table:**

Agilent Xcalibur Ruby Gemini diffractometer	4929 independent reflections
Radiation source: Enhance (Cu) X-ray Source	4100 reflections with *I* > 2σ(*I*)
Graphite monochromator	*R*_int_ = 0.028
Detector resolution: 10.5081 pixels mm^-1^	θ_max_ = 75.6°, θ_min_ = 2.9°
ω scans	*h* = −42→42
Absorption correction: multi-scan (*CrysAlis PRO*; Agilent, 2012)	*k* = −8→11
*T*_min_ = 0.788, *T*_max_ = 1.000	*l* = −20→21
9307 measured reflections	

### Refinement

**Table:**

Refinement on *F*^2^	Primary atom site location: structure-invariant direct methods
Least-squares matrix: full	Secondary atom site location: difference Fourier map
*R*[*F*^2^ > 2σ(*F*^2^)] = 0.034	Hydrogen site location: inferred from neighbouring sites
*wR*(*F*^2^) = 0.093	H atoms treated by a mixture of independent and constrained refinement
*S* = 1.03	*w* = 1/[σ^2^(*F*_o_^2^) + (0.0514*P*)^2^ + 0.4711*P*] where *P* = (*F*_o_^2^ + 2*F*_c_^2^)/3
4929 reflections	(Δ/σ)_max_ = 0.004
308 parameters	Δρ_max_ = 0.43 e Å^−^^3^
0 restraints	Δρ_min_ = −0.35 e Å^−^^3^

### Special details

**Table:**

Experimental. ^1^H NMR (400 MHz, CDCl_3_): δ (ppm) 7.87-7.71 (1H, m), 7.46-7.31 (4H, m), 7.09-7.07 (1H, m), 6.54-6.48 (1H, m), 3.53 (2H, s), 3.45 (2H, s), 2.89 (2H, s), 1.38 (1H, s), 1.13 (6H, s). ^13^C NMR (CDCl_3_): δ 29.4, 30.9, 31.1, 31.9, 31.2, 34.7, 35.1, 35.5, 53.9, 109.7, 109.9, 120.2, 122.1, 122.5, 122.7, 123.1, 123.3, 129.4, 129.7, 131.2, 133.0, 135.6, 142.8, 151.7, 152.6, 152.8.
Geometry. All e.s.d.'s (except the e.s.d. in the dihedral angle between two l.s. planes) are estimated using the full covariance matrix. The cell e.s.d.'s are taken into account individually in the estimation of e.s.d.'s in distances, angles and torsion angles; correlations between e.s.d.'s in cell parameters are only used when they are defined by crystal symmetry. An approximate (isotropic) treatment of cell e.s.d.'s is used for estimating e.s.d.'s involving l.s. planes.
Refinement. Refinement of *F*^2^ against ALL reflections. The weighted *R*-factor *wR* and goodness of fit *S* are based on *F*^2^, conventional *R*-factors *R* are based on *F*, with *F* set to zero for negative *F*^2^. The threshold expression of *F*^2^ > σ(*F*^2^) is used only for calculating *R*-factors(gt) *etc*. and is not relevant to the choice of reflections for refinement. *R*-factors based on *F*^2^ are statistically about twice as large as those based on *F*, and *R*- factors based on ALL data will be even larger.

### Fractional atomic coordinates and isotropic or equivalent isotropic displacement parameters (Å^2^)

**Table:**

	*x*	*y*	*z*	*U*_iso_*/*U*_eq_	
Br	0.630352 (8)	0.53882 (3)	0.543344 (15)	0.03435 (9)	
O1	0.52147 (5)	0.66100 (17)	0.36987 (11)	0.0338 (3)	
N1	0.70366 (6)	0.3018 (2)	0.53797 (13)	0.0370 (4)	
N2	0.65609 (7)	0.1320 (2)	0.53017 (14)	0.0380 (4)	
N3	0.56938 (7)	0.7887 (2)	0.19074 (15)	0.0406 (5)	
H3B	0.5490 (9)	0.765 (3)	0.1989 (18)	0.033 (7)*	
N4	0.57538 (6)	0.78517 (19)	0.36118 (13)	0.0315 (4)	
C1	0.72063 (8)	0.4328 (3)	0.52039 (19)	0.0447 (6)	
H1A	0.6998	0.4684	0.4614	0.067*	
H1B	0.7492	0.4142	0.5227	0.067*	
H1C	0.7244	0.5039	0.5653	0.067*	
C2	0.72791 (8)	0.2049 (3)	0.60366 (16)	0.0401 (5)	
C3	0.77297 (9)	0.1991 (4)	0.66449 (19)	0.0541 (7)	
H3A	0.7929	0.2717	0.6682	0.065*	
C4	0.78664 (11)	0.0803 (4)	0.7190 (2)	0.0620 (9)	
H4A	0.8171	0.0697	0.7600	0.074*	
C5	0.75708 (12)	−0.0240 (4)	0.7155 (2)	0.0615 (8)	
H5A	0.7679	−0.1020	0.7554	0.074*	
C6	0.71279 (11)	−0.0174 (3)	0.6561 (2)	0.0542 (7)	
H6A	0.6929	−0.0892	0.6541	0.065*	
C7	0.69805 (8)	0.1004 (3)	0.59819 (16)	0.0402 (5)	
C8	0.66112 (7)	0.2518 (2)	0.49703 (15)	0.0323 (4)	
C9	0.62557 (7)	0.3270 (2)	0.42009 (14)	0.0292 (4)	
C10	0.60873 (7)	0.2663 (2)	0.33589 (15)	0.0300 (4)	
H10A	0.6198	0.1769	0.3297	0.036*	
C11	0.57614 (7)	0.3331 (2)	0.26044 (14)	0.0292 (4)	
C12	0.55997 (7)	0.4627 (2)	0.27251 (15)	0.0294 (4)	
H12A	0.5371	0.5086	0.2224	0.035*	
C13	0.57612 (6)	0.5266 (2)	0.35517 (14)	0.0264 (4)	
C14	0.60895 (7)	0.4577 (2)	0.42851 (14)	0.0276 (4)	
C15	0.55815 (8)	0.2676 (2)	0.16780 (16)	0.0358 (5)	
C16	0.59482 (11)	0.1859 (3)	0.15917 (19)	0.0543 (7)	
H16A	0.6042	0.1043	0.1996	0.081*	
H16B	0.6200	0.2490	0.1744	0.081*	
H16C	0.5835	0.1524	0.0980	0.081*	
C17	0.52108 (12)	0.1646 (4)	0.1534 (2)	0.0693 (11)	
H17A	0.4978	0.2157	0.1592	0.104*	
H17B	0.5328	0.0888	0.1979	0.104*	
H17C	0.5089	0.1232	0.0938	0.104*	
C18	0.54015 (8)	0.3820 (3)	0.09601 (15)	0.0363 (5)	
H18A	0.5131	0.4223	0.0925	0.054*	
H18B	0.5336	0.3400	0.0387	0.054*	
H18C	0.5622	0.4573	0.1108	0.054*	
C19	0.55554 (7)	0.6638 (2)	0.36324 (13)	0.0275 (4)	
C20	0.55836 (9)	0.9201 (2)	0.3754 (2)	0.0426 (6)	
H20A	0.5362	0.9011	0.3947	0.064*	
H20B	0.5448	0.9743	0.3199	0.064*	
H20C	0.5827	0.9751	0.4212	0.064*	
C21	0.61458 (7)	0.7918 (2)	0.35017 (17)	0.0326 (5)	
C22	0.65527 (8)	0.8083 (3)	0.42439 (18)	0.0414 (5)	
H22A	0.6574	0.8092	0.4818	0.050*	
C23	0.69305 (8)	0.8234 (3)	0.4155 (2)	0.0511 (7)	
H23A	0.7211	0.8336	0.4664	0.061*	
C24	0.68929 (8)	0.8235 (3)	0.3315 (2)	0.0507 (7)	
H24A	0.7151	0.8333	0.3251	0.061*	
C25	0.64900 (8)	0.8098 (3)	0.2569 (2)	0.0435 (6)	
H25A	0.6474	0.8101	0.2000	0.052*	
C26	0.60991 (7)	0.7951 (2)	0.26395 (17)	0.0346 (5)	
C27	0.56379 (9)	0.7866 (3)	0.10141 (18)	0.0464 (6)	
H27A	0.5323	0.7794	0.0588	0.070*	
H27B	0.5795	0.7047	0.0945	0.070*	
H27C	0.5758	0.8743	0.0905	0.070*	

### Atomic displacement parameters (Å^2^)

**Table:**

	*U*^11^	*U*^22^	*U*^33^	*U*^12^	*U*^13^	*U*^23^
Br	0.03690 (13)	0.03680 (13)	0.02700 (13)	0.00268 (9)	0.01323 (10)	−0.00251 (9)
O1	0.0297 (7)	0.0358 (8)	0.0387 (8)	−0.0016 (6)	0.0186 (7)	−0.0021 (7)
N1	0.0310 (9)	0.0397 (10)	0.0355 (10)	0.0022 (8)	0.0118 (8)	0.0008 (9)
N2	0.0409 (10)	0.0310 (9)	0.0377 (10)	0.0037 (8)	0.0151 (9)	0.0023 (8)
N3	0.0332 (10)	0.0481 (12)	0.0406 (11)	−0.0087 (9)	0.0175 (9)	−0.0048 (9)
N4	0.0269 (9)	0.0257 (8)	0.0416 (10)	0.0007 (7)	0.0159 (8)	−0.0004 (8)
C1	0.0359 (12)	0.0481 (14)	0.0495 (15)	−0.0062 (11)	0.0196 (12)	−0.0017 (12)
C2	0.0389 (12)	0.0445 (13)	0.0324 (12)	0.0115 (10)	0.0132 (10)	0.0003 (10)
C3	0.0398 (14)	0.072 (2)	0.0381 (14)	0.0112 (13)	0.0085 (12)	−0.0019 (14)
C4	0.0498 (16)	0.083 (2)	0.0357 (14)	0.0283 (16)	0.0059 (13)	0.0053 (15)
C5	0.071 (2)	0.0625 (19)	0.0414 (15)	0.0313 (17)	0.0189 (15)	0.0140 (14)
C6	0.0675 (19)	0.0453 (15)	0.0460 (15)	0.0195 (14)	0.0237 (15)	0.0108 (12)
C7	0.0439 (13)	0.0377 (12)	0.0349 (12)	0.0119 (10)	0.0153 (11)	0.0026 (10)
C8	0.0334 (11)	0.0304 (10)	0.0312 (11)	0.0043 (9)	0.0138 (9)	−0.0007 (9)
C9	0.0270 (10)	0.0282 (10)	0.0307 (11)	−0.0011 (8)	0.0124 (9)	0.0003 (8)
C10	0.0302 (10)	0.0243 (9)	0.0355 (11)	−0.0010 (8)	0.0155 (9)	−0.0012 (8)
C11	0.0321 (10)	0.0241 (9)	0.0313 (11)	−0.0072 (8)	0.0150 (9)	−0.0027 (8)
C12	0.0292 (10)	0.0267 (10)	0.0294 (10)	−0.0019 (8)	0.0115 (9)	0.0023 (8)
C13	0.0246 (9)	0.0239 (9)	0.0302 (10)	−0.0031 (8)	0.0125 (8)	−0.0007 (8)
C14	0.0279 (10)	0.0289 (10)	0.0249 (10)	−0.0031 (8)	0.0117 (8)	−0.0022 (8)
C15	0.0483 (13)	0.0268 (10)	0.0300 (11)	−0.0073 (10)	0.0167 (10)	−0.0028 (9)
C16	0.082 (2)	0.0392 (13)	0.0388 (14)	0.0177 (14)	0.0262 (15)	0.0001 (11)
C17	0.089 (2)	0.071 (2)	0.0360 (14)	−0.053 (2)	0.0204 (16)	−0.0103 (14)
C18	0.0436 (12)	0.0350 (11)	0.0307 (11)	0.0006 (10)	0.0179 (10)	−0.0003 (9)
C19	0.0249 (9)	0.0289 (10)	0.0242 (10)	0.0005 (8)	0.0080 (8)	−0.0015 (8)
C20	0.0406 (12)	0.0276 (11)	0.0617 (16)	0.0050 (10)	0.0261 (12)	0.0003 (11)
C21	0.0271 (10)	0.0223 (9)	0.0484 (13)	−0.0001 (8)	0.0179 (10)	0.0013 (9)
C22	0.0332 (12)	0.0383 (12)	0.0450 (14)	−0.0049 (10)	0.0123 (11)	0.0063 (11)
C23	0.0282 (12)	0.0508 (15)	0.0623 (18)	−0.0053 (11)	0.0116 (12)	0.0077 (13)
C24	0.0316 (12)	0.0470 (14)	0.077 (2)	−0.0034 (11)	0.0282 (13)	0.0031 (14)
C25	0.0407 (13)	0.0375 (12)	0.0617 (16)	−0.0055 (10)	0.0320 (13)	−0.0044 (12)
C26	0.0310 (11)	0.0244 (9)	0.0477 (13)	−0.0018 (8)	0.0182 (10)	−0.0021 (9)
C27	0.0515 (15)	0.0427 (13)	0.0443 (14)	−0.0125 (12)	0.0221 (12)	−0.0076 (11)

### Geometric parameters (Å, º)

**Table:**

Br—C14	1.901 (2)	C12—C13	1.388 (3)
O1—C19	1.232 (3)	C12—H12A	0.9500
N1—C8	1.373 (3)	C13—C14	1.389 (3)
N1—C2	1.379 (3)	C13—C19	1.510 (3)
N1—C1	1.456 (3)	C15—C18	1.526 (3)
N2—C8	1.310 (3)	C15—C17	1.527 (3)
N2—C7	1.391 (3)	C15—C16	1.545 (4)
N3—C26	1.365 (3)	C16—H16A	0.9800
N3—C27	1.447 (3)	C16—H16B	0.9800
N3—H3B	0.81 (3)	C16—H16C	0.9800
N4—C19	1.340 (3)	C17—H17A	0.9800
N4—C21	1.450 (3)	C17—H17B	0.9800
N4—C20	1.467 (3)	C17—H17C	0.9800
C1—H1A	0.9800	C18—H18A	0.9800
C1—H1B	0.9800	C18—H18B	0.9800
C1—H1C	0.9800	C18—H18C	0.9800
C2—C7	1.394 (4)	C20—H20A	0.9800
C2—C3	1.401 (4)	C20—H20B	0.9800
C3—C4	1.388 (5)	C20—H20C	0.9800
C3—H3A	0.9500	C21—C22	1.383 (3)
C4—C5	1.395 (5)	C21—C26	1.406 (3)
C4—H4A	0.9500	C22—C23	1.387 (3)
C5—C6	1.376 (5)	C22—H22A	0.9500
C5—H5A	0.9500	C23—C24	1.379 (4)
C6—C7	1.412 (4)	C23—H23A	0.9500
C6—H6A	0.9500	C24—C25	1.375 (4)
C8—C9	1.484 (3)	C24—H24A	0.9500
C9—C14	1.393 (3)	C25—C26	1.414 (3)
C9—C10	1.395 (3)	C25—H25A	0.9500
C10—C11	1.395 (3)	C27—H27A	0.9800
C10—H10A	0.9500	C27—H27B	0.9800
C11—C12	1.396 (3)	C27—H27C	0.9800
C11—C15	1.531 (3)		
			
C8—N1—C2	106.0 (2)	C18—C15—C11	111.07 (18)
C8—N1—C1	128.4 (2)	C17—C15—C11	108.6 (2)
C2—N1—C1	125.5 (2)	C18—C15—C16	108.3 (2)
C8—N2—C7	104.4 (2)	C17—C15—C16	109.0 (3)
C26—N3—C27	122.4 (2)	C11—C15—C16	110.6 (2)
C26—N3—H3B	117 (2)	C15—C16—H16A	109.5
C27—N3—H3B	119 (2)	C15—C16—H16B	109.5
C19—N4—C21	123.86 (18)	H16A—C16—H16B	109.5
C19—N4—C20	119.04 (18)	C15—C16—H16C	109.5
C21—N4—C20	117.02 (18)	H16A—C16—H16C	109.5
N1—C1—H1A	109.5	H16B—C16—H16C	109.5
N1—C1—H1B	109.5	C15—C17—H17A	109.5
H1A—C1—H1B	109.5	C15—C17—H17B	109.5
N1—C1—H1C	109.5	H17A—C17—H17B	109.5
H1A—C1—H1C	109.5	C15—C17—H17C	109.5
H1B—C1—H1C	109.5	H17A—C17—H17C	109.5
N1—C2—C7	105.7 (2)	H17B—C17—H17C	109.5
N1—C2—C3	131.4 (3)	C15—C18—H18A	109.5
C7—C2—C3	123.0 (3)	C15—C18—H18B	109.5
C4—C3—C2	115.7 (3)	H18A—C18—H18B	109.5
C4—C3—H3A	122.1	C15—C18—H18C	109.5
C2—C3—H3A	122.1	H18A—C18—H18C	109.5
C3—C4—C5	122.0 (3)	H18B—C18—H18C	109.5
C3—C4—H4A	119.0	O1—C19—N4	122.7 (2)
C5—C4—H4A	119.0	O1—C19—C13	119.92 (19)
C6—C5—C4	122.0 (3)	N4—C19—C13	117.37 (17)
C6—C5—H5A	119.0	N4—C20—H20A	109.5
C4—C5—H5A	119.0	N4—C20—H20B	109.5
C5—C6—C7	117.2 (3)	H20A—C20—H20B	109.5
C5—C6—H6A	121.4	N4—C20—H20C	109.5
C7—C6—H6A	121.4	H20A—C20—H20C	109.5
N2—C7—C2	110.2 (2)	H20B—C20—H20C	109.5
N2—C7—C6	129.8 (3)	C22—C21—C26	121.5 (2)
C2—C7—C6	120.0 (3)	C22—C21—N4	119.1 (2)
N2—C8—N1	113.7 (2)	C26—C21—N4	119.1 (2)
N2—C8—C9	124.9 (2)	C21—C22—C23	120.3 (3)
N1—C8—C9	121.4 (2)	C21—C22—H22A	119.8
C14—C9—C10	118.6 (2)	C23—C22—H22A	119.8
C14—C9—C8	122.40 (19)	C24—C23—C22	119.0 (3)
C10—C9—C8	119.00 (19)	C24—C23—H23A	120.5
C9—C10—C11	121.9 (2)	C22—C23—H23A	120.5
C9—C10—H10A	119.0	C25—C24—C23	121.4 (2)
C11—C10—H10A	119.0	C25—C24—H24A	119.3
C10—C11—C12	117.3 (2)	C23—C24—H24A	119.3
C10—C11—C15	121.96 (19)	C24—C25—C26	120.8 (3)
C12—C11—C15	120.7 (2)	C24—C25—H25A	119.6
C13—C12—C11	122.3 (2)	C26—C25—H25A	119.6
C13—C12—H12A	118.8	N3—C26—C21	121.4 (2)
C11—C12—H12A	118.8	N3—C26—C25	121.8 (2)
C12—C13—C14	118.62 (19)	C21—C26—C25	116.8 (2)
C12—C13—C19	119.05 (19)	N3—C27—H27A	109.5
C14—C13—C19	122.21 (19)	N3—C27—H27B	109.5
C13—C14—C9	121.17 (19)	H27A—C27—H27B	109.5
C13—C14—Br	119.78 (16)	N3—C27—H27C	109.5
C9—C14—Br	119.01 (16)	H27A—C27—H27C	109.5
C18—C15—C17	109.2 (2)	H27B—C27—H27C	109.5
			
C8—N1—C2—C7	0.1 (2)	C12—C13—C14—Br	−177.74 (14)
C1—N1—C2—C7	177.6 (2)	C19—C13—C14—Br	−1.6 (3)
C8—N1—C2—C3	178.1 (3)	C10—C9—C14—C13	0.4 (3)
C1—N1—C2—C3	−4.3 (4)	C8—C9—C14—C13	179.08 (19)
N1—C2—C3—C4	−176.7 (3)	C10—C9—C14—Br	178.12 (15)
C7—C2—C3—C4	1.0 (4)	C8—C9—C14—Br	−3.2 (3)
C2—C3—C4—C5	−2.2 (4)	C10—C11—C15—C18	−154.3 (2)
C3—C4—C5—C6	1.9 (5)	C12—C11—C15—C18	26.3 (3)
C4—C5—C6—C7	−0.2 (5)	C10—C11—C15—C17	85.6 (3)
C8—N2—C7—C2	−0.3 (3)	C12—C11—C15—C17	−93.8 (3)
C8—N2—C7—C6	−178.8 (3)	C10—C11—C15—C16	−34.1 (3)
N1—C2—C7—N2	0.1 (3)	C12—C11—C15—C16	146.5 (2)
C3—C2—C7—N2	−178.1 (2)	C21—N4—C19—O1	−177.7 (2)
N1—C2—C7—C6	178.7 (2)	C20—N4—C19—O1	5.7 (3)
C3—C2—C7—C6	0.5 (4)	C21—N4—C19—C13	1.1 (3)
C5—C6—C7—N2	177.4 (3)	C20—N4—C19—C13	−175.5 (2)
C5—C6—C7—C2	−0.9 (4)	C12—C13—C19—O1	82.7 (3)
C7—N2—C8—N1	0.4 (3)	C14—C13—C19—O1	−93.4 (2)
C7—N2—C8—C9	177.9 (2)	C12—C13—C19—N4	−96.1 (2)
C2—N1—C8—N2	−0.3 (3)	C14—C13—C19—N4	87.7 (2)
C1—N1—C8—N2	−177.8 (2)	C19—N4—C21—C22	−98.6 (3)
C2—N1—C8—C9	−177.9 (2)	C20—N4—C21—C22	78.2 (3)
C1—N1—C8—C9	4.6 (4)	C19—N4—C21—C26	87.6 (3)
N2—C8—C9—C14	112.4 (3)	C20—N4—C21—C26	−95.7 (3)
N1—C8—C9—C14	−70.3 (3)	C26—C21—C22—C23	−2.4 (4)
N2—C8—C9—C10	−68.9 (3)	N4—C21—C22—C23	−176.1 (2)
N1—C8—C9—C10	108.4 (2)	C21—C22—C23—C24	0.8 (4)
C14—C9—C10—C11	0.4 (3)	C22—C23—C24—C25	0.4 (4)
C8—C9—C10—C11	−178.28 (19)	C23—C24—C25—C26	0.1 (4)
C9—C10—C11—C12	−1.6 (3)	C27—N3—C26—C21	−177.6 (2)
C9—C10—C11—C15	179.03 (19)	C27—N3—C26—C25	4.3 (4)
C10—C11—C12—C13	2.0 (3)	C22—C21—C26—N3	−175.4 (2)
C15—C11—C12—C13	−178.63 (19)	N4—C21—C26—N3	−1.7 (3)
C11—C12—C13—C14	−1.2 (3)	C22—C21—C26—C25	2.8 (3)
C11—C12—C13—C19	−177.46 (18)	N4—C21—C26—C25	176.5 (2)
C12—C13—C14—C9	0.0 (3)	C24—C25—C26—N3	176.5 (2)
C19—C13—C14—C9	176.10 (18)	C24—C25—C26—C21	−1.6 (4)

### Hydrogen-bond geometry (Å, º)

**Table:**

*D*—H···*A*	*D*—H	H···*A*	*D*···*A*	*D*—H···*A*
N3—H3*B*···O1^i^	0.81 (3)	2.35 (3)	3.038 (3)	143 (3)
C4—H4*A*···Br^ii^	0.95	2.98	3.719 (3)	135
C12—H12*A*···O1^i^	0.95	2.37	3.287 (3)	163

Symmetry codes: (i) −*x*+1, *y*, −*z*+1/2; (ii) −*x*+3/2, *y*−1/2, −*z*+3/2.
